# Nutritional risk screening and analysis of factors influencing nutritional risk in patients with stable chronic obstructive pulmonary disease

**DOI:** 10.12669/pjms.41.12.12215

**Published:** 2025-12

**Authors:** Najuan Cui, Yongliang Cui, Qinghua Wu, Shaohua Wang, Wei Wang

**Affiliations:** 1 Najuan Cui, Department of Spleen and Stomach Diseases, Beijing Hospital of Integrated Traditional Chinese and Western Medicine, Hospital of Integrated Traditional Chinese and Western Medicine Beijing University of Chinese Medicine, Beijing 100039, China; 2 Yongliang Cui, Department of Cardiology, First Hospital of Tsinghua University, Beijing 100016, China; 3 Qinghua Wu, Department of Respiratory, Beijing Hospital of Integrated Traditional Chinese and Western Medicine, Hospital of Integrated Traditional Chinese and Western Medicine Beijing University of Chinese Medicine, Beijing 100039, China; 4 Shaohua Wang, Department of Spleen and Stomach Diseases, Beijing Hospital of Integrated Traditional Chinese and Western Medicine, Hospital of Integrated Traditional Chinese and Western Medicine Beijing University of Chinese Medicine, Beijing 100039, China; 5 Wei Wang, Department of Spleen and Stomach Diseases, Beijing Hospital of Integrated Traditional Chinese and Western Medicine, Hospital of Integrated Traditional Chinese and Western Medicine Beijing University of Chinese Medicine, Beijing 100039, China

**Keywords:** Chronic obstructive pulmonary disease, Stable disease, Nutritional risk, Factor

## Abstract

**Objective:**

To assess nutritional status and influencing factors in stable chronic obstructive pulmonary disease (COPD) patients.

**Methodology:**

This retrospective study of 280 stable COPD patients from Beijing Hospital of Integrated Traditional Chinese and Western Medicine and the First Affiliated Hospital of Tsinghua University (June 2022 to June 2024). Collected data included demographics, body mass index (BMI), smoking history, lung function, nutritional risk, six-minute walk distance (6MWD) test, COPD Assessment Test (CAT) scores, and traditional Chinese medicine (TCM) syndrome patterns. Statistical analysis compared nutritionally normal (NN) and at-risk (NAR) groups.

**Results:**

Significant differences (p< 0.05) between NN and NAR groups included age, BMI, smoking history (active/passive), CAT scores, lung function, 6MWD, and TCM syndromes (spleen/kidney deficiency, qi/yin deficiency). Logistic regression identified key factors: sex, age, BMI, smoking (history/index), CAT/6MWD/lung function classifications, and TCM patterns (spleen/kidney involvement, qi/blood stasis).

**Conclusion:**

Nutritional risk in stable COPD is significantly influenced by age, BMI, smoking, disease severity (CAT/lung function), physical capacity (6MWD), and TCM syndromes (deficiency/stasis patterns). These factors should guide nutritional interventions.

## INTRODUCTION

Chronic obstructive pulmonary disease (COPD) is a chronic wasting disease. As the disease progresses, patients are often at risk of malnutrition.^1^,^2^ Malnutrition can lead to weakened immune function, triggering acute exacerbations of COPD and negatively affecting patient prognosis. It can also result in reduced respiratory muscle strength and endurance, leading to respiratory muscle fatigue. Therefore, malnutrition is an independent risk factor influencing the prognosis of patients with COPD.^3^,^4^ Implementing appropriate nutritional interventions for patients with stable COPD is essential to reducing the frequency of acute exacerbations. This study aimed to support the nutritional intervention of patients with COPD by screening for nutritional status and analyzing factors influencing nutritional risk in patients with stable COPD.

## METHODOLOGY

This s was a retrospective cohort study, including 280 patients with stable COPD who were admitted to Beijing Hospital of Integrated Traditional Chinese and Western Medicine and the First Affiliated Hospital of Tsinghua University from June 2022 to June 2024. According to the “COPD Diagnosis and Treatment Guidelines (Revised Edition 2013)”, COPD is diagnosed when FEV1/FVC<70% after inhalation of bronchodilators, and other respiratory diseases are excluded. The stable period is defined as when symptoms such as coughing and expectoration are stable or significantly improved compared to the acute exacerbation period. Nutritional risk grouping: Screening was conducted using the Mini Nutritional Assessment Form (MNA-SF). Those with MNA-SF<11 were classified as the nutritional risk group (NAR, 125 cases), and those with ≥11 were classified as the normal nutrition group (NN, 155 cases).

### Ethical Approval

The study was approved by the Ethics Committee of Beijing Hospital of Integrated Traditional Chinese and Western Medicine (No.: ZXEC-KT-2022-02-P03, Date: May 12, 2022), and all patients signed the informed consent form.

### Inclusion criteria:

- Patients who meet the diagnostic criteria for COPD and anxiety and depression disorders.^6^
- Patients without cognitive impairment and able to communicate with the scale and questionnaires normally.
- Patients who themselves and their families have no objection to the content of this study and signed the informed consent form.

### Exclusion criteria:

- Patients with respiratory system-related diseases other than COPD.
- Patients with severe damage to vital organs of the body such as the heart, brain, liver, and kidney.
- Patients with mental disorders other than anxiety and depression.
- Patients with malignant tumors or patients undergoing malignant tumor surgery.
- Patients already on bronchodilator therapy, malignancy and bronchiectasis.

The following data were collected by trained researchers through standardized forms:

### Baseline characteristics:

Gender, age (stratification: ≤60 years old, 61-70 years old, >70 years old), BMI (grading: <18.5, 18.5- 24,24 -28, ≥28), smoking history [passive smoking history (PSH), active smoking history (ASH), daily smoking amount (DCC), smoking index (SI)].

### Western medical examination:

Pulmonary function classification (Grade I-IV, based on the percentage of FEV1 in the predicted value). CAT score (0-40 points, assessing quality of life, classification: mild 0-10, moderate 11-20, severe 21-30, extremely severe 31-40). A six minutes’ walk distance (6MWD, classification: severe <238 m, moderate 238-388 m, mild > 388 m).

### Traditional Chinese Medicine (TCM) assessment:

Based on the “Diagnostic Criteria for TCM Syndromes of COPD (2011 Edition)”, data from the four diagnostic methods were collected, with a focus on analyzing the distribution of syndrome elements such as spleen deficiency, lung deficiency, and kidney deficiency.

### Statistical Analysis:

Descriptive statistics: Continuous variables are described by mean ± standard deviation (normal distribution) or median (IQR). The power of test / confidence interval is 95%. Categorical variables are expressed as frequency and percentage. Intergroup comparison: Independent sample t-test was used for continuous variables with normal distribution, and Wilcoxon rank sum test was used for non-normal distribution. Categorical variables were tested using the χ^2^ test or Fisher’s exact probability method. Multivariate analysis: Firstly, variables were screened through univariate Logistic regression (α=0.10), and then the multivariate Logistic regression model (α=0.05) was included to calculate the odds ratio (OR) and 95% confidence interval (CI).

## RESULTS

A total of 280 patients with stable COPD were included. 44.64% (125 cases) had nutritional risk (NAR group), and 55.36% (155 cases) had normal nutrition (NN group). The MNA-SF score ranged from 5.00 to 15.00, with a mean of 11.12±2.25 and a median of 11.00 (Table-I).

**Table-I:** MNA-SF score and nutritional risk distribution.

Parameter	Total
Sample size	280
Mean±SD	11.12±2.25
Median (IQR)	11.00(3.00)
NN, N(%)	155(55.36%)
NAR, N(%)	125(44.64%)

### Comparison of baseline characteristics between groups:

### Demography and smoking history:

The age in the NAR group was significantly higher (68.82±8.74 vs. 66.54±8.62 years old, P=0.0296), but there was no difference in age stratification (P=0.2282). The passive smoking history (PSH), active smoking history (ASH), daily smoking amount (DCC), and smoking index (SI) in the NAR group were significantly higher than those in the NN group (P<0.05), especially the proportion of those with SI>400 reached 45.6% (Table-II).

**Table-II:** Inter-group comparison of indicators related to smoking history.

Parameter	NN group	NAR group	P-value
PSH			0.0433*
N (Missing)	151(4)	123(2)	
Absent, N(%)	115(76.16)	80(65.04)	
Present, N(%)	36(23.84)	43(34.96)	
ASH			0.0125*
N (Missing)	155(0)	125(0)	
Absent, N(%)	89(57.42)	53(42.40)	
Present, N(%)	66(42.58)	72(57.60)	
DCC [cigarettes/d]			0.0146*
N (Missing)	63(92)	72(53)	
Mean±SD	16.68±9.99	19.92±9.30	
Median (IQR)	15.00(10.00)	20.00(5.00)	
Q1, Q3	10.00,20.00	15.00,20.00	
Min, Max	1.00,60.00	5.00,60.00	
SI classification			0.0008*
N (Missing)	155(0)	125(0)	
0, N(%)	92(59.35)	53(42.40)	
≤400, N(%)	24(15.48)	15(12.00)	
>400, N(%)	39(25.16)	57(45.60)	

### Pulmonary function and functional status:

The pulmonary function classification in the NAR group was higher (Grade II-IV accounted for 96.8% vs. 72.26%, P<0.0001), CAT score (17.82±7.25 vs. 13.61±7.44, P<0.0001), and six minutes walking distance (6MWD). 335.44±81.66 cm vs. 406.43±83.02 cm, P<0.0001) were both significantly worse than the NN group (Table-III-IV). (3) Distribution of TCM syndrome elements: In the NAR group, the proportions of syndrome elements such as spleen deficiency (84.8% vs. 39.35%), kidney deficiency (46.4% vs. 33.55%), and lung deficiency (94.4% vs. 62.58%) were significantly higher (P<0.05) (Table-V).

**Table-III:** Pulmonary function classification and CAT score.

Parameter	NN group	NAR group	P-value
Pulmonary function classification			<0.0001*
Grade-I, N(%)	43(27.74)	4(3.20)	
Grade-II, N(%)	42(27.10)	34(27.20)	
Grade-III, N(%)	50(32.26)	53(42.40)	
Grade-IV, N(%)	20(12.90)	34(27.20)	
CAT score			<0.0001*
Mean±SD	13.61±7.44	17.82±7.25	
CAT classification			0.0003*
Mild, N(%)	64(41.29)	25(20.00)	
Moderate, N(%)	54(34.84)	55(44.00)	
Severe, N(%)	36(23.23)	41(32.80)	
Very Severe, N(%)	1(0.65)	4(3.20)	

**Table-IV:** Distribution and between-group comparison of 6MWD and classification.

Parameter	NN group	NAR group	P-value
6MWD			<0.0001*
Mean±SD	406.43±83.02	335.44±81.66	
6MWD classification			<0.0001*
Grade-1 (severe), N(%)	5(6.10)	9(9.89)	
Grade-2 (moderate), N(%)	20(24.39)	53(58.24)	
Grade-3 (mild), N(%)	57(69.51)	29(31.87)	

**Table-V:** Distribution of key TCM syndrome elements.

Syndrome element	NN group	NAR group	P-value
Spleen	61(39.35)	106(84.80)	<0.0001*
Kidney	52(33.55)	58(46.40)	0.0286*
Spleen deficiency	59(38.06)	105(84.00)	<0.0001*
Lung deficiency	97(62.58)	118(94.40)	<0.0001*
Kidney deficiency	51(32.90)	56(44.80)	0.0417*
Yin deficiency	11(7.10)	26(20.80)	0.0008*
Qi deficiency	18(11.61)	32(25.60)	0.0024*

### Analysis of Influencing Factors:

### Univariate regression:

Gender, BMI grade, smoking history, lung function grade, and TCM syndrome elements (spleen deficiency, lung deficiency, kidney deficiency, etc.) were significantly associated with nutritional risk (P<0.05).

### Multivariate regression:

Independent risk factors: low BMI (0<BMI<18.5, OR=3.275), lung function Grade (Grade II-IV, OR=6.788-19.263), Spleen deficiency syndrome element (OR=7.024, P<0.0001). Protective factors: BMI 24-28 kg/m^2^ (OR=0.376, P=0.0425) (Table-VI).

**Table-VI:** Multivariate Logistic regression results.

Parameter	Reference range	Category	P-value	OR (95% CI)
BMI classification	18.5≤BMI<24	BMI≥28	0.0033	0.057(0.008,0.385)
	18.5≤BMI<24	24≤BMI<28	0.0425	0.376(0.146,0.967)
	18.5≤BMI<24	0<BMI<18.5	0.3930	3.275(0.215,49.812)
Pulmonary function classification	Grade-I	Grade-IV	0.0004	19.263(3.770,98.412)
	Grade-I	Grade-III	0.0006	17.806(3.454,91.796)
	Grade-I	Grade-II	0.0181	6.788(1.386,33.234)
Disease location-spleen	Absent	Present	<0.0001	7.024(2.737,18.026)

## DISCUSSION

This study focused on the influencing factors of nutritional status in patients with stable chronic obstructive pulmonary disease (COPD). Through univariate and multivariate analyses, it was found that there were significant differences (P<0.05) between the nutritional risk group (NAR) and the normal nutrition group (NN) in indicators such as age, weight, BMI, lung function classification, TCM syndrome elements (spleen and kidney related syndrome elements, spleen deficiency, lung deficiency, kidney deficiency, etc.), CAT score, and six minutes walking distance (6MWD). Multivariate analysis further indicated that low BMI (0<BMI<18.5), higher lung function classification (Grades II-IV), and spleen deficiency syndrome element were independent risk factors for nutritional risk. Among them, spleen deficiency syndrome element increased the risk of malnutrition by 6.024 times. The nutritional risk in the BMI 24-28 kg/m^2^ group was 37.6% lower than that in the normal weight group (OR=0.624). Furthermore, the smoking index (SI) in the NAR group was significantly higher than that in the NN group, suggesting that long-term smoking may exacerbate malnutrition by affecting the function of the digestive system.

In this study, 44.7% of COPD patients had nutritional risks, which was consistent with the prevalence range of 10%-45% reported in previous literature,^1^,^5^ confirming the prevalence of malnutrition in COPD patients. This study found that the higher the lung function grade (the more severe the condition), the higher the nutritional risk, which is consistent with the results of many international studies.^6^-^8^ The mechanism may be related to the hypermetabolic state caused by the disease, digestive dysfunction (such as gastrointestinal congestion and dysbiosis), and weakened immune function. For example, frequent acute exacerbations of COPD can intensify inflammatory responses (such as elevated C-reactive protein), further consuming the body’s energy reserves.^9^-^11^

In this study, the smoking index (SI) of the NAR group was significantly higher, which was consistent with the result of a domestic study that included 500 COPD patients.^12^ Nicotine can cause gastritis and inhibit the absorption of nutrients by interfering with the regulation of the digestive system by the autonomic nervous system,^13^,^14^ which explains the positive correlation between the amount of smoking and nutritional risk. This study confirmed for the first time through quantitative analysis that spleen deficiency syndrome element is an independent risk factor for nutritional risk in patients with COPD, and the risk increase is up to six times. This is highly consistent with the view in traditional Chinese medicine theory that “the spleen and stomach are the foundation of the body after birth” - spleen deficiency leads to abnormal transportation and transformation of food and water, insufficient production of qi and blood, and subsequently causes malnutrition.^15^-^17^ In contrast, Western research mostly focuses on biological indicators. This study provides a new dimension for nutritional assessment from the perspective of TCM syndrome elements.

For the first time, traditional Chinese medicine syndrome elements (such as spleen deficiency and kidney deficiency) were included in the multivariate analysis of nutritional risk in COPD, and their independent influencing effects were found, providing evidence for the assessment of nutritional status through the combination of traditional Chinese and Western medicine. It has been confirmed that within the normal range of BMI (18.5-24 kg/m^2^), a higher BMI (24-28 kg/m^2^) has a protective effect on nutritional risk, which is consistent with the clinical observation that “moderate overweight in COPD patients may have a better prognosis”.^18^,^19^

It is recommended that routine nutritional screening (e.g., MNA-SF) should be implemented for COPD patients, with focused attention on those exhibiting low BMI, severe lung dysfunction, or spleen deficiency syndrome to enable early intervention. Smokers require concurrent smoking cessation support and digestive function assessment to break the “smoking-digestive dysfunction-malnutrition” cycle. Traditional Chinese Medicine (TCM) syndrome patterns, particularly spleen deficiency, can supplement nutritional risk prediction and inform personalized interventions, such as spleen-strengthening and qi-boosting therapies to enhance nutrient absorption. The advantage of this study lies in integrating the clinical indicators of Western medicine with the syndrome elements of traditional Chinese medicine, and adopting a multi-dimensional analysis method to reveal the pathological mechanisms of traditional Chinese medicine that have not been concerned by traditional biomedicine. Functional status indicators such as CAT score and 6MWD were included to comprehensively evaluate the association between nutritional status and the quality of life of patients.

### Limitations:

It includes a small number of patients. In the future, multi-center and large-sample studies need to be carried out to verify the association between TCM syndrome elements and nutritional risks, and to explore the influence of gender and regional differences.

## CONCLUSIONS

In summary, the nutritional status of patients with stable COPD is influenced by multiple factors, including age, smoking history, pulmonary function, and TCM syndrome elements. These factors are all associated with the affected population’s quality of life. For at-risk elderly patients with COPD, individualized nutritional interventions can improve their nutritional status, pulmonary function, and exercise tolerance.

### Authors’ Contributions:

**NC:** Concept, study design, literature search and manuscript writing.

**QW:** Data collection, data analysis, interpretation and critical review.

**SW** and **WW:** Revision of manuscript and validation.

All authors have read, approved the final manuscript and are responsible for the integrity of the study.
